# Effects of Dog-assisted Therapy in Anxiety Symptoms of Female Adolescents With Eating Disorders: A Controlled Trial

**DOI:** 10.62641/aep.v53i6.1919

**Published:** 2025-12-17

**Authors:** Beatriz Martínez Núñez, Javier Fernández Sánchez, Ana Myriam Lavín Pérez, Luis-Lucio Lobato Rincón, Manuel Fonseca García, Israel González García, Montserrat Graell Berna, Daniel Collado Mateo

**Affiliations:** ^1^Psychiatry and Psychology Department, Niño Jesús University Children’s Hospital, 28009 Madrid, Spain; ^2^Sports Science Research Centre, Rey Juan Carlos University, 28943 Fuenlabrada, Madrid, Spain; ^3^Department of Experimental Psychology, Cognitive Processes and Speech Therapy, Faculty of Psychology, Complutense University of Madrid, 28223 Pozuelo de Alarcón, Madrid, Spain; ^4^Animal-Assisted Interventions Office, King Juan Carlos University, 28933 Móstoles, Madrid, Spain; ^5^Perroterapia Non-Governmental Association, 28523 Rivas-Vaciamadrid, Madrid, Spain

**Keywords:** animal-assisted interventions, eating disorders, anorexia, bulimia, dog therapy

## Abstract

**Background:**

Eating disorders are a cluster of challenging psychiatric and medical conditions that constitute a major problem in adolescents and young adults. Animal-assisted interventions are currently a promising complementary therapy with great development in the hospital field.

**Methods:**

The study is a non-randomized clinical trial designed with two groups under assessment. The first group consisted of 15 female patients at a Day Hospital diagnosed with eating disorders who received animal-assisted therapy. The second group had a sample of 15 patients from the same hospital with similar diagnoses and matched characteristics to the intervention group. Anxiety, depression, eating symptoms, grip strength, and health-related quality of life were analyzed both previously and after the intervention.

**Results:**

ANOVA results showed a significant between-group reduction in the state anxiety levels (*p* = 0.011, ηp^2^ = 0.211), with a significant decrease in the intervention group (*p* = 0.003). Interpersonal distrust (*p* = 0.042) and fear of maturity (*p* = 0.012) subscales in the Eating Disorder Inventory (EDI2) questionnaire had larger improvement when comparing pre- and post-measures in the intervention group. A similar trend was observed for the rest of the eating symptoms subscales and grip strength in the group treated with the animal-assisted intervention.

**Conclusion:**

This study suggests that dog-assisted therapy may be an effective complementary intervention for reducing state anxiety in adolescents with eating disorders. Given the role of anxiety in the maintenance of anorexia nervosa, targeting this symptom could have therapeutic benefits. Additionally, improvements in interpersonal distrust and maturity fears were observed, highlighting the potential impact of animal-assisted therapy on specific psychological factors associated with eating disorders. These findings support the inclusion of dog-assisted interventions as part of multidisciplinary treatment approaches, although further research with larger samples is needed to confirm these effects.

**Clinical trial registration:**

clinicaltrials.gov (NCT04869423).

## Introduction

Eating disorders (EDs) are complex psychiatric conditions characterized by preoccupation with weight, food, and body image [1]. They typically emerge during adolescence or early adulthood and result in both physical and psychological impairments [1]. Among EDs, anorexia nervosa and bulimia nervosa are the most studied, with lifetime prevalence estimates of up to 4% in women and 1% in men [2], with an overall prevalence around 176 per 100,000 individuals [3]. The consequences of EDs extend beyond eating behaviors. Anxiety and panic attacks are highly prevalent among individuals with EDs, often linked to the development of generalized and social anxiety disorders, mood dysregulation, self-esteem, and perfectionism [4]. Depression, also common in EDs, often involves persistent sadness, hopelessness, and a loss of interest in daily activities with an unfortunate lack of efficacy of antidepressants in this population [5].

Current treatments for EDs are multidisciplinary, including nutritional rehabilitation, psychotherapy, and pharmacotherapy. Despite the efficacy of these approaches, complementary therapies such as animal-assisted interventions are emerging as promising adjunct treatments for EDs. Animal-assisted therapies (ATT) are designed to promote psychosocial well-being through interaction with animals and contribute to the enhancement of social skills and promote increased socialization with peers in different clinical populations, particularly in interventions involving dogs [6]. The physical contact with the animal has been identified as main factor for AAT effectiveness, distracting from pain and negative feelings, reducing the stress and enhancing the patient’s perception of illness through the reduction of the fear of being judged by others [7].

Despite its potential, the use of AAT in the treatment of EDs remains limited, with existing studies varying widely in methodology and design [8]. Qualitative reports suggest potential benefits such as improved cognitive flexibility, self-confidence, and emotional expression. Some patients also describe enhanced body image and increased willingness to engage in healthy activities when participating in AAT [8]. However, research specifically addressing anxiety in adolescents with EDs is lacking, despite the strong role of anxiety in symptom maintenance and is particularly relevant given the high prevalence of anxiety as a comorbid condition in this population. Therefore, this pilot study aims to assess the impact of AAT on anxiety and Health Related Quality of Life (HRQoL) variables in adolescents with ED.

## Methods

### Design

This pilot study used a parallel-group, non-randomized clinical trial to evaluate AAT in adolescents with eating disorders versus a control group receiving standard care. Conducted at the Psychiatry and Psychology Department of Niño Jesús University Children’s Hospital in Madrid, Spain, the study followed SPIRIT 2013 guidelines, adhered to the Declaration of Helsinki, and received ethical approval from the Ethics Research Committee of the Niño Jesús University Children’s Hospital (R-0007/21, March 2021). The trial was registered at ClinicalTrials.gov (NCT04869423). Participants were assigned to either AAT plus standard care or standard care alone. Evaluators were blinded, and confidentiality was ensured through anonymous IDs. Pre- and post-intervention assessments measured mental and physical health outcomes. The study also complied with Spain’s Organic Law 3/2018 on Data Protection and Digital Rights Guarantee.

### Participants

The participants were adolescents under the age of 18 who were treated at the Day Hospital of the Psychiatry and Psychology Department at Niño Jesús Children’s Hospital. They were diagnosed with an eating disorder, such as anorexia nervosa, bulimia nervosa, binge eating disorder, or other eating disorders, according to DSM-5 criteria. The following inclusion criteria were set: (1) be willing to participate, (2) be available to attend the sessions, and (3) sign the written informed consent by both the adolescent and their legal guardian. Exclusion criteria included: (1) allergies, (2) phobias to dogs, and (3) any history of aggressive behavior toward animals.

Participants were recruited voluntarily between April and October 2021. Enrolment was based on opportunity and conditioned by medical criteria and the availability of participants to attend the sessions.

### Intervention

The intervention lasted seven weeks, with adolescents participating in 50-minute AAT sessions once per week. These sessions were integrated with the hospital’s standard care, allowing participants to continue their usual treatments without interruption. To ensure effective supervision, therapy was conducted in small groups of four participants. Adolescents discharged from the hospital could continue the intervention, while those with allergies or aggressive behavior toward dogs were excluded from the experimental group.

Each session consisted of three phases: introduction to the therapy dog (5 minutes), interactive activities targeting therapeutic goals (40 minutes), and a closing phase (5 minutes). The core activity involved teaching basic dog training techniques through interactive exercises designed to encourage physical movement while addressing therapeutic objectives. These included strengthening the patient-dog bond, reducing anxiety, improving mood, enhancing social skills, increasing impulse control, and promoting self-esteem. The structured approach allowed participants to progress toward their goals over time.

In the closing phase, participants said goodbye to the therapy dog, reinforcing the bond established during the session. Sessions were supervised by a psychologist and two dog handlers, who ensured the dogs’ welfare and maintained a safe, hygienic environment. Handlers monitored the dogs’ physical and emotional well-being, and activities were gradually made more interactive as participants developed trust and familiarity with the dogs.

The intervention aimed to maximize physical contact with the therapy dogs, enhancing both mental and physical health outcomes. The control group continued with standard care, which could include pharmacological and non-pharmacological treatments provided by the Spanish public health system, without participation in AAT.

### Variables and Measurements

The evaluations were conducted at the Psychiatry and Clinical Psychology Department of the Niño Jesús University Children’s Hospital following the completion of the interventions.

#### Sociodemographic Characteristics

The assessment protocol included the collection of sociodemographic information from participants, including age and gender. Furthermore, clinical data were documented, including the specific diagnosis of the eating and body mass index (BMI).

#### Anxiety Measurement

To assess the anxiety levels of adolescents, the State-Trait Anxiety Inventory for Children (STAI-C) was employed [9]. This Spanish-validated instrument includes two 20-item scales: one measuring state anxiety, reflecting temporary anxiety at a specific moment, and the other measuring trait anxiety, representing a more stable personal characteristic [10]. Each item on the STAI-C was rated on a three-point Likert scale, ranging from 1 to 3. Consequently, scores for each sub-scale ranged from 20 to 60, with higher scores indicating higher levels of anxiety [9].

The STAI-C also provides centile and point scores. Centile scores show an individual’s relative standing compared to normative data, with scores above the 85th percentile indicating clinically significant anxiety. Point scores, or “punts”, are raw totals from item responses, reflecting the overall level of anxiety experienced by the participant. This dual scoring system allows both clinical interpretation relative to peers and a direct measure of individual anxiety severity, supporting comprehensive assessment in research and practice [9].

#### Strength Measurement

Participants’ strength was measured through an isometric maximum strength test utilizing a handgrip dynamometer (Takei TKK 5401 Digital Handgrip Dynamometer, Tokyo, Japan) model used before in adolescents [11]. In this protocol, participants were instructed to exert maximum pressure on the dynamometer with their dominant hand while keeping their arms fully extended [12]. The validity of this assessment method was well-established in clinical settings, where handgrip strength served as a reliable indicator of overall physical strength and health status [13].

#### Health-related Quality of Life Measurement

HRQoL measurement was evaluated using the Kidscreen-10 Index, a validated instrument comprising 10 items aimed at assessing the well-being and health of children and adolescents [14]. This tool allowed for the comparison of individual results with general population norms, facilitating the classification of scores as either “normal” (within the average range) or “sensitive” (below average).

The Kidscreen-10 demonstrated robust reliability, evidenced by an internal consistency coefficient (Cronbach’s α) of 0.82 and a test-retest reliability coefficient (r) of 0.73 [Intraclass Correlation Coefficien (ICC) = 0.72]. The scoring range spanned from 10 to 50, with higher scores reflecting superior HRQoL [15]. Additionally, the Kidscreen-10 employed was the Spanish version translated and validated from the original [16].

#### Depression Measurement

The Children Depression Inventory (CDI) was used to measure depressive symptoms in children and adolescents. This widely recognized tool includes 27 items rated on a 3-point Likert scale ranging from 0 (no symptoms) to 2 (severe symptoms) [17]. For this research, the study applied the Spanish-validated version of the CDI [18]. The total possible score on the CDI spans from 0 to 54, with higher scores reflecting greater severity of depression [17]. The CDI is known for its robust psychometric properties, including internal consistency (Cronbach’s α) between 0.75 and 0.94, and a test-retest reliability coefficient of 0.84 [18].

#### Eating Disorder Symptoms Measurement

The Eating Disorder Inventory-2 (EDI-2) was used to assess the progression of symptoms in anorexia nervosa and bulimia nervosa. It contains 91 items across 11 subscales: Drive for Thinness, Bulimia, Body Dissatisfaction, Ineffectiveness, Perfectionism, Interpersonal Distrust, Interoceptive Awareness, Maturity Fears, Asceticism, Impulse Regulation, and Social Insecurity. Responses are rated on a Likert scale from 0 (never) to 5 (usually), with higher scores indicating more severe symptoms [19].

In this study, subscale scores were calculated by summing item responses, and these were combined to produce an overall total score, which can be converted into percentiles using normative data. Percentiles allow comparison to reference populations and help identify individuals at higher risk or needing intervention [19]. Although no universal cut-off exists, higher scores generally reflect greater severity. The EDI-2 has demonstrated strong reliability and internal consistency, with Cronbach’s alpha values often reaching 0.70, and has been validated across cultural contexts, including Spanish populations, supporting its clinical and research use [20].

### Statistical Analysis

Statistical analyses were performed using IBM SPSS version 25.0 (SPSS, Inc., Chicago, IL, USA). Continuous variables were presented as means and standard deviations for both baseline and post-intervention assessments. Normality was assessed with the Shapiro-Wilk test. Baseline group comparisons used the Student’s *t*-test for normally distributed variables or the Mann-Whitney U test for non-normal data. Correlations at baseline were examined with Pearson’s or Spearman’s coefficients, depending on data distribution. To assess the effects of the AAT intervention, repeated measures ANOVA evaluated interaction effects between time (baseline vs. post-intervention) and group (AAT vs. control), with the sphericity assumption verified [21]. Baseline differences in the total CDI score and its self-esteem dimension were included as covariates in the ANOVA. A *p*-value < 0.05 indicated statistical significance. Post-hoc pairwise comparisons applied the Bonferroni correction, and partial eta squared (η^2^ₚ) was reported.

## Results

### Participants’ Characteristics

Regarding participants’ characteristics, a total of 30 adolescents were finally included in the study. The participants’ mean age was 15.84 years (SD = 0.99), while their mean BMI was 18.75 kg/m^2^ (SD = 1.89). There were no significant between-group differences at baseline for these two variables. Most of the participants were in the weight restoration phase of recovery from Anorexia Nervosa Restricting Type, except for one participant in recovery from Bulimia Nervosa Purging Type. Recovery was defined based on medical criteria (partial weight restoration), clinical improvement in ED symptoms, and engagement in multidisciplinary treatment at the Day Hospital. Although it was not an inclusion criterion, all participants in the study were female. Detailed information on each intervention group can be found in Table 1.

**Table 1.** **Participants’ characteristics**.

		Animal-assisted therapy group (n = 15)	Control group (n = 15)
		Mean ± SD	Mean ± SD
Age		16.053 ± 0.911	15.611 ± 1.037
Body mass index		18.781 ± 1.872	18.711 ± 1.971
		n (%)	n (%)
Diagnosis			
	Anorexia nervosa-restricting type	15 (100)	14 (93.33)
	Bulimia nervosa purging type	0 (0)	1 (6.67)

Some participants received Selective Serotonin Reuptake Inhibitors (SSRIs) for anxiety or depression and nutritional supplements, but there were no significant differences between the two groups regarding the proportion of patients receiving medication. No participants were undergoing specific physical therapy interventions beyond standard medical recommendations for movement encouragement in eating disorder treatment.

### Correlations Between Variables at Baseline 

Table 2 shows the correlations between the variables assessed prior to the intervention. The Spearman test showed that grip strength was positively and significantly associated with HRQoL (*p* = 0.032). In addition, HRQoL was inversely associated with both, state (*p *= 0.002) and trait anxiety (*p *= 0.035).

**Table 2.** **Correlations between the variables before the intervention**.

Variables	Anxiety state	Anxiety trait	Children depression	HRQoL	Grip strength
Anxiety state		0.451^*^	–0.007	–0.561^**^	–0.264
Anxiety trait	0.451^*^		–0.024	–0.408^*^	–0.119
Children depression	–0.007	–0.024		–0.147	0.164
HRQoL	–0.561^**^	–0.408^*^	–0.147		0.413^*^
Grip strength	–0.264	–0.119	0.164	0.413^*^	

HRQoL, Health-related quality of life; ^*^Significant correlation with *p* < 0.05; ^**^Significant correlation with *p* < 0.01.

### Animal-assisted Therapy Effects

Table 3 shows the results obtained in both groups on the variables state anxiety, trait anxiety, grip strength, HRQoL, and self-esteem and dysphoria. Significant differences between the AAT group and the control group at baseline were observed in the CDI total score and the self-esteem dimension. On the other hand, there were no differences in terms of age, clinical diagnosis, anxiety, eating disorder severity, HRQoL, or grip strength. This ensures comparability between groups before the intervention.

**Table 3.** **Effects of animal-assisted therapy in anxiety, strength, depression and health-related quality of life**.

	Animal-assisted therapy group mean (SD)		Control group mean (SD)		Interaction *p*-value (Effect Size, ηp²)
	Baseline	After	Baseline	After	
Anxiety state	44.47 (7.65)	37.27 (9.47)	45.60 (8.76)	47.00 (10.02)	0.011 (0.211) ^ƚ^
Anxiety state centile	90.40 (13.78)	68.00 (34.41)	85.21 (25.46)	88.93 (25.28)	0.007 (0.235)^ƚ^
Anxiety state punts	81.93 (13.68)	63.13 (26.69)	82.53 (19.89)	83.33 (24.73)	0.016 (0.189)^ƚ^
Anxiety trait	49.00 (6.93)	46.87 (8.85)	52.00 (3.72)	51.33 (7.55)	0.601 (0.010)
Anxiety trait centile	91.80 (15.03)	81.33 (27.97)	96.33 (5.77)	90.67 (27.75)	0.558 (0.012)
Anxiety trait punts	85.93 (15.09)	75.67 (24.75)	91.00 (9.83)	86.27 (19.57)	0.451 (0.020)^ƚ^
Grip strength D	17.39 (5.62)	19.17 (4.83)	18.27 (5.61)	18.10 (5.90)	0.690 (0.113)^ƚ^
Grip Strength ND	16.52 (4.95)	17.08 (4.46)	17.55 (5.36)	17.13 (5.20)	0.248 (0.047)
CDI total score	25.13 (2.53)*	24.47 (2.42)	21.73 (4.50)	21.47 (2.72)	0.073 (0.114)
CDI self-esteem	10.20 (1.27)*	9.80 (1.42)	8.60 (1.92)	8.67 (2.26)	0.478 (0.019)
CDI dysphoria	14.93 (2.05)	14.67 (1.88)	13.13 (3.29)	12.80 (2.30)	0.927 (0.000)
Kidscreen-10	28.00 (7.98)	30.15 (8.36)	26.93 (5.54)	26.73 (7.80)	0.381 (0.030)

SD, Standard deviation; ES, effect size; D, dominant; ND, not dominant; CDI, Children Depression Inventory; *Significant differences between Animal Assisted Therapy Group and Control Group at baseline; ^ƚ^ Significant differences between before and after measurements in the animal-assisted therapy group.

The ANOVA found a significant effect of the interaction between time and group on state anxiety (F = 7.503, *p* = 0.011, ηp^2^ = 0.211), as well as on its centile (F = 8.578, *p* = 0.007, ηp^2^ = 0.235) and score (F = 6.540, *p* = 0.016, ηp^2^ = 0.189). Pairwise comparisons revealed a significant reduction in state anxiety scores in the AAT group after the intervention (*p* = 0.003). Although no significant interaction effect was found for grip strength (dominant hand), pairwise comparisons showed a significant improvement in the AAT group after the intervention (*p* = 0.021). No significant interaction effects were observed for trait anxiety, non-dominant hand grip strength, or the self-esteem and dysphoria dimensions of the CDI scale (all *p* > 0.05).

**Fig. 1.**
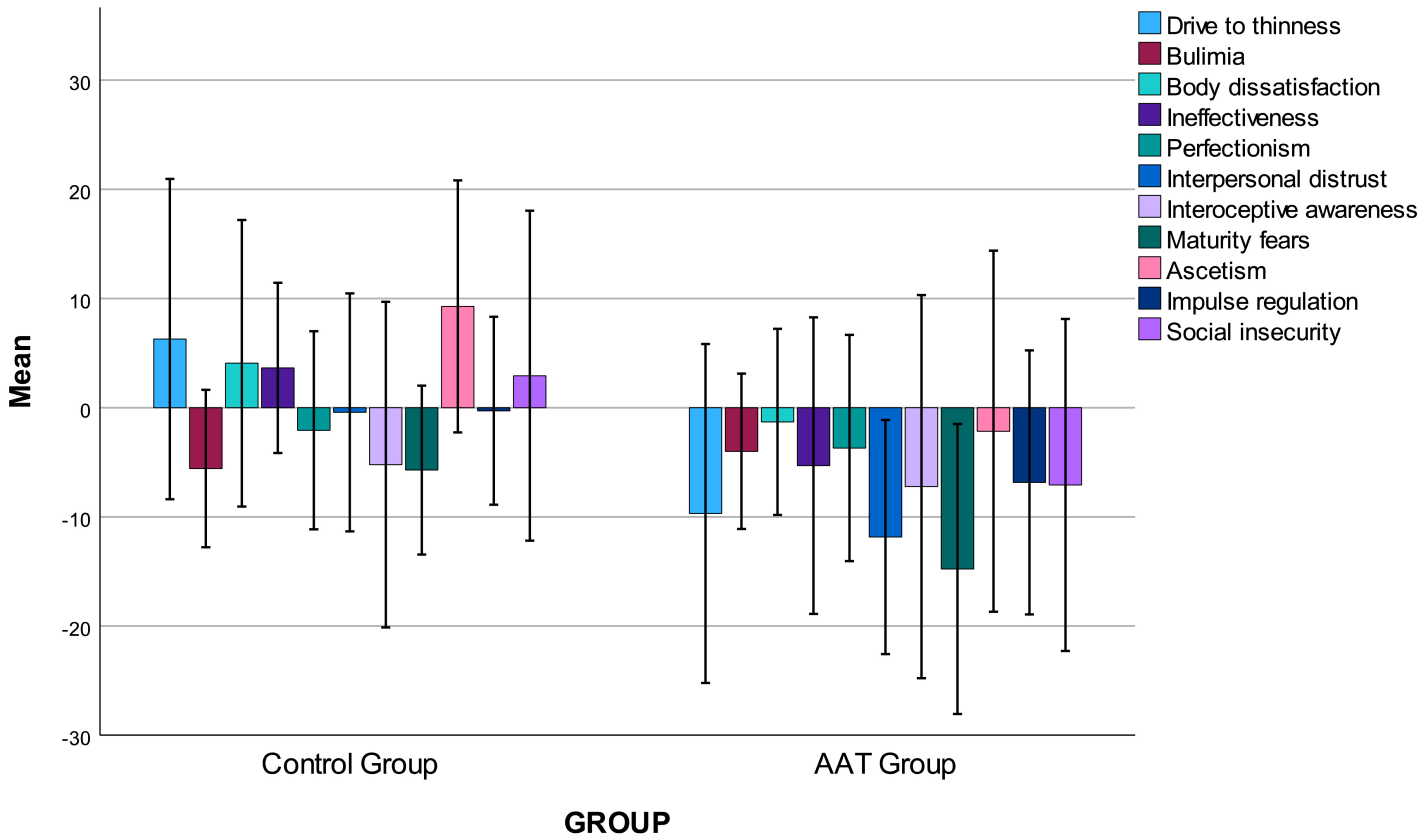
**Changes in anorexia nervosa and bulimia nervosa symptoms in the AAT group and control groups pre- and post-intervention**. Note: Error bars: ± 2 SE.

As shown in Fig. 1, the AAT group demonstrated significant reductions in interpersonal distrust (*p* = 0.042) and maturity fears (*p* = 0.012) compared to baseline. Baseline scores indicated moderate to severe symptomatology in both groups, consistent with clinical levels of eating disorder pathology. No statistically significant differences were found in total post-intervention. However, significant improvements were observed in the interpersonal distrust (*p* = 0.042) and maturity fears (*p* = 0.012) subscales in the intervention group. These findings suggest that AAT may contribute to alleviating specific psychological aspects related to eating disorder symptomatology, rather than producing a general reduction in symptoms.

## Discussion

This pilot study suggests that AAT may significantly reduce state anxiety in adolescents with eating disorders, accompanied by improvements in interpersonal distrust and maturity fears. These findings indicate that AAT may target specific psychological dimensions rather than producing a general reduction in symptomatology. Such results are consistent with previous studies on eating disorder psychopathology [8], as well as with improvements found in other populations reducing anxiety and depression while improving overall wellbeing [22]. However, in contrast with prior research that found promising results in HRQoL following animal-assisted interventions [23], our study did not observe significant effects in this domain. This discrepancy may reflect differences in sample characteristics, intervention duration, or outcome measures.

The reduction in state anxiety is clinically meaningful given its high prevalence among individuals with eating disorders and its role in symptom maintenance [24]. In this regard, the relationship between anxiety and eating disorder symptoms is suggested to be bi-directional so these two variables may establish a dangerous vicious cycle [4]. Thus, based on these findings, incorporating interventions that reduce anxiety may enhance treatment outcomes. Among the interventions aimed at reducing anxiety, this study is, to the best of our knowledge, the first to analyze and suggest anxiety reduction through a dog-assisted therapy program. Furthermore, this controlled and novel research identified a potential association between the intervention and a decrease in symptoms related to these disorders, such as interpersonal distrust and maturity fears. These symptom reductions may be particularly relevant, as scientific literature has identified interpersonal distrust as a significant predictor of subsequent depression [25]. Therefore, the decrease in these symptoms appears to be linked to reductions in both anxiety and somatization [25].

The mechanisms underlying the psychological effects of AAT are likely to be multifactorial. Thus, interactions with animals can increase oxytocin, dopamine, and endorphins, which potentially explaining observed reductions in anxiety and improvements in mood [26]. Additionally, the absence of perceived judgment from animals may provide a sense of safety and acceptance, particularly relevant for individuals with eating disorders [7].

Concerning the physical variables, a significant improvement in dominant-hand grip strength in the group that received the dog-assisted intervention. These increases achieved by the AAT are crucial as they are an indicative variable of the nutritional status of the participants, presenting up to 16–70% less strength than the healthy population [27]. Furthermore, grip strength is a relevant variable in this population, and is associated with their HRQoL, as the current correlation shows in accordance with the scientific literature in other populations [13]. These improvements might be indirectly related to mood enhancement resulting from human–animal interaction. A better emotional state and motivation may encourage participants to engage in more active daily behaviors, thereby contributing to better physical performance [28]. Importantly, the activities in therapy sessions were not specifically designed to improve upper-limb strength, as they occurred once weekly over seven weeks with limited physical demand. Thus, changes could also be attributable to daily lifestyle modifications.

Several limitations must be considered. The sample size may have limited statistical power, preventing the detection of significant differences across all variables. As a pilot study, the primary aim was to assess feasibility and preliminary efficacy, which was supported by observed reductions in anxiety and related symptoms. A double-blind design was not feasible, and although some outcomes improved, significant interaction effects were not consistently observed. These factors suggest that findings should be interpreted as exploratory.

Despite these limitations, the findings highlight the potential of AAT as an adjunctive treatment for EDs, particularly in addressing anxiety and related symptoms. Future studies should explore the long-term effects of AAT, considering variables such as treatment adherence, symptom remission, and overall HRQoL. In addition, transdiagnostic factors such as internal awareness or perfectionism could be explored or treated in specific AAT interventions, investigating the extent of these interventions.

## Conclusion

This study indicates that dog-assisted therapy may effectively reduce state anxiety in adolescent females with eating disorders compared to standard care. Improvements were also seen in specific symptoms, including maturity fears and interpersonal distrust, as well as in physical health, reflected by increased grip strength. These results suggest AAT’s potential as a complementary treatment, addressing both psychological and physical aspects of eating disorders. Reducing state anxiety, a key factor in the maintenance of ED pathology, is particularly important as it is linked to better treatment outcomes and quality of life. Integrating AAT into multidisciplinary programs could enhance therapeutic engagement and help alleviate core symptoms.

## Availability of Data and Materials

The datasets analyzed in the current study are not publicly available due to privacy and ethical restrictions, but are available from the corresponding author on reasonable request, after approval by the Ethics Committee.

## References

[1] Barakat S, McLean SA, Bryant E, Le A, Marks P, National Eating Disorder Research Consortium (2023). Risk factors for eating disorders: findings from a rapid review. *Journal of Eating Disorders*.

[2] van Eeden AE, van Hoeken D, Hoek HW (2021). Incidence, prevalence and mortality of anorexia nervosa and bulimia nervosa. *Current Opinion in Psychiatry*.

[3] Santomauro DF, Melen S, Mitchison D, Vos T, Whiteford H, Ferrari AJ (2021). The hidden burden of eating disorders: an extension of estimates from the Global Burden of Disease Study 2019. *The Lancet. Psychiatry*.

[4] Trompeter N, Dârvariu Ș, Brieva-Toloza AV, Opitz MC, Rabelo-da-Ponte FD, Sharpe H (2025). The prospective relationship between anxiety symptoms and eating disorder symptoms among adolescents: a systematic review and meta-analysis of a bi-directional relationship. *European Child & Adolescent Psychiatry*.

[5] Mischoulon D, Eddy KT, Keshaviah A, Dinescu D, Ross SL, Kass AE (2011). Depression and eating disorders: treatment and course. *Journal of Affective Disorders*.

[6] Shoesmith E, Surr C, Ratschen E (2023). Animal-assisted and robotic animal-assisted interventions within dementia care: A systematic review. *Dementia (London, England)*.

[7] Shen RZZ, Xiong P, Chou UI, Hall BJ (2018). “We need them as much as they need us”: A systematic review of the qualitative evidence for possible mechanisms of effectiveness of animal-assisted intervention (AAI). *Complementary Therapies in Medicine*.

[8] Fennig MW, Weber E, Santos B, Fitzsimmons-Craft EE, Wilfley DE (2022). Animal-assisted therapy in eating disorder treatment: A systematic review. *Eating Behaviors*.

[9] Spielberger CD, Edwards CD, Montouri J, Lushene R (1973). *State-Trait Anxiety Inventory for Children*.

[10] Salvador GP, Navarro MD (1994). Estudio psicométrico del State-Trait Anxiety Inventory for Children (STAIC). *Psicológica: Revista de metodología y psicología experimental*.

[11] Martínez-Torres J, Gallo-Villegas JA, Aguirre-Acevedo DC (2022). Normative values for handgrip strength in Colombian children and adolescents from 6 to 17 years of age: estimation using quantile regression. *Jornal De Pediatria*.

[12] Etemadi S, Sun GX, Leung SP, Siddique A, Cooper S, Ezenwa NC (2021). The Sit Up Squat Stand test and Hand Grip Strength: What is the role of tests of muscle power in risk assessment in Anorexia Nervosa?. *European Eating Disorders Review: the Journal of the Eating Disorders Association*.

[13] Vaishya R, Misra A, Vaish A, Ursino N, D’Ambrosi R (2024). Hand grip strength as a proposed new vital sign of health: a narrative review of evidences. *Journal of Health, Population, and Nutrition*.

[14] Ravens-Sieberer U, Gosch A, Abel T, Auquier P, Bellach BM, Bruil J (2001). Quality of life in children and adolescents: a European public health perspective. *Sozial- Und Praventivmedizin*.

[15] Ravens-Sieberer U, Erhart M, Rajmil L, Herdman M, Auquier P, Bruil J (2010). Reliability, construct and criterion validity of the KIDSCREEN-10 score: a short measure for children and adolescents’ well-being and health-related quality of life. *Quality of Life Research: an International Journal of Quality of Life Aspects of Treatment, Care and Rehabilitation*.

[16] Aymerich M, Berra S, Guillamón I, Herdman M, Alonso J, Ravens-Sieberer U (2005). Development of the Spanish version of the KIDSCREEN, a health-related quality of life instrument for children and adolescents. *Gaceta Sanitaria*.

[17] Kovacs M (2003). *Children’s Depression Inventory (CDI)*.

[18] Davanzo P, Kerwin L, Nikore V, Esparza C, Forness S, Murrelle L (2004). Spanish translation and reliability testing of the Child Depression Inventory. *Child Psychiatry and Human Development*.

[19] Garner DM, Olmstead MP, Polivy J (1983). Development and validation of a multidimensional eating disorder inventory for anorexia nervosa and bulimia. *International Journal of Eating Disorders*.

[20] Urzua M, Castro R, Lillo O, Leal P (2009). Evaluation of eating disorders: psychometric properties of EDI-2 in students 13 to 18 years old. *Revista Chilena de Nutrición*.

[21] Blanca MJ, Arnau J, García-Castro FJ, Alarcón R, Bono R (2023). Non-normal Data in Repeated Measures ANOVA: Impact on Type I Error and Power. *Psicothema*.

[22] Antolín Esteve L, López-Mases P, Bartolomé Del Pino LE, Lázaro E (2025). Animal-Assisted Therapy for the Management of Anxiety in the Hospital Setting: A Systematic Review. *Holistic Nursing Practice*.

[23] Correale C, Borgi M, Collacchi B, Falamesca C, Gentile S, Vigevano F (2022). Improving the Emotional Distress and the Experience of Hospitalization in Children and Adolescent Patients Through Animal Assisted Interventions: A Systematic Review. *Frontiers in Psychology*.

[24] Sander J, Moessner M, Bauer S (2021). Depression, Anxiety and Eating Disorder-Related Impairment: Moderators in Female Adolescents and Young Adults. *International Journal of Environmental Research and Public Health*.

[25] Gómez Penedo JM, Meglio M, Flückiger C, Wienicke FJ, Breunese J, Menchetti M (2025). Interpersonal problems as a predictor of treatment outcome in adult depression: An individual participant data meta-analysis. *Clinical Psychology Review*.

[26] Beetz A, Uvnäs-Moberg K, Julius H, Kotrschal K (2012). Psychosocial and psychophysiological effects of human-animal interactions: the possible role of oxytocin. *Frontiers in Psychology*.

[27] Rosa-Caldwell ME, Eddy KT, Rutkove SB, Breithaupt L (2023). Anorexia nervosa and muscle health: A systematic review of our current understanding and future recommendations for study. *The International Journal of Eating Disorders*.

[28] Cameron DS, Bertenshaw EJ, Sheeran P (2018). Positive affect and physical activity: Testing effects on goal setting, activation, prioritisation, and attainment. *Psychology & Health*.

